# Learning-Based Image Damage Area Detection for Old Photo Recovery

**DOI:** 10.3390/s22218580

**Published:** 2022-11-07

**Authors:** Tien-Ying Kuo, Yu-Jen Wei, Po-Chyi Su, Tzu-Hao Lin

**Affiliations:** 1 Department of Electrical Engineering, National Taipei University of Technology, Taipei 10608, Taiwan; 2 Department of Computer Science and Information Engineering, National Central University, Taoyuan City 32001, Taiwan

**Keywords:** deep learning, damage area detection, damaged old photo

## Abstract

Most methods for repairing damaged old photos are manual or semi-automatic. With these methods, the damaged region must first be manually marked so that it can be repaired later either by hand or by an algorithm. However, damage marking is a time-consuming and labor-intensive process. Although there are a few fully automatic repair methods, they are in the style of end-to-end repairing, which means they provide no control over damaged area detection, potentially destroying or being unable to completely preserve valuable historical photos to the full degree. Therefore, this paper proposes a deep learning-based architecture for automatically detecting damaged areas of old photos. We designed a damage detection model to automatically and correctly mark damaged areas in photos, and this damage can be subsequently repaired using any existing inpainting methods. Our experimental results show that our proposed damage detection model can detect complex damaged areas in old photos automatically and effectively. The damage marking time is substantially reduced to less than 0.01 s per photo to speed up old photo recovery processing.

## 1. Introduction

Old photos can often contain various levels of damage caused by human improper storage or environmental factors that deteriorate the integrity of photos. Fortunately, digital image processing technology can be applied to recover the content of these photos to its original state. The existing recovery methods for damaged old photos can be divided into non-automatic and automatic processes according to whether human intervention is required. The non-automatic methods can be further subdivided into manual and semi-automatic methods. Manual recovery is made through a variety of image editing tools, such as Photoshop or GIMP [1], to recover damaged photos based on user knowledge. The semi-automatic method manually marks the damaged areas on the photos and then applies the inpainting methods [2,3] to recover the contents of these locations. The mentioned works focused on the design of repair methods. For example, Li et al. [2] modified the confidence computation, strategy matching, and filling scheme to improve the inpainting method. Zhao et al. [3] proposed an inpainting model based on the generative adversarial network (GAN) and gated convolution [4]. With their methods, in addition to the damaged photos as the input, additional damage masks should be specified before inputting into the model. Non-automatic methods, while providing good recovery results, require physically marking the damaged areas in the photos, taking a lot of time and effort.

The automatic method does not have the aforementioned problems as it does not require any additional information in the process of restoring damaged old photos. Works [5,6] have used deep learning techniques to develop automatic methods that can be applied to a wider variety of photo content and types of damage. Wan et al. [5] designed a model based on the architecture of variational autoencoder (VAE) [7]. They used an encoder model to first obtain the feature vectors representing the input photo in the latent space, then used the latent restoration network to remove the damage and noisy components embedded in the feature vectors, and finally the feature vectors were reverted back to the recovered photo. Liu et al. [6] designed two modules: latent class-attribute restoration (LCR) and dynamic condition-guided restoration (DCR). LCR first analyzes the four class attributes of smoothness, clarity, connectivity, and completeness in the photo to repair the global defects and then uses multiple DCRs in series to process the local defects to restore the details in the photo. Although the automatic method can reduce the processing time for restoring damaged old photos, the results generated are not satisfactory. For example, in [5,6], some textures and objects were removed from the recovered photos because they were mistakenly treated as noise or damage, and some undamaged areas in the photos were also modified, which is undoubtedly a problem for preserving the integrity of the photo content.

In order to improve these shortcomings, we propose a method by which to automatically detect damaged areas in old photos and use the detection results to guide inpainting methods to automatically recover the original content of these areas. In general, damaged area detection involves finding damaged areas in objects, such as steel structures [8], murals [9], photos [10,11], frescoes [12], and pavements [13,14,15,16,17,18,19], through algorithms. The methods for detecting damaged areas can be divided into traditional algorithms and deep learning algorithms depending on the development method.

Damage detection methods [9,10,11,12] were developed using traditional image processing techniques. Jaidilert et al. [9] used seeded region growing [20] and morphology to detect cracks. Bhuvaneswari et al. [10] combined a bilateral filter and Haar wavelet transform to detect scratch damage in images. The Hough transformation was used in [11] to detect line cracks in images. Cornelis et al. [12] believed that the luminance value of cracks is low, so the top-hat transformation of morphology was used to find cracks with a low luminance value. The damage detection methods mentioned above are not effective in detecting irregular damage areas and can only detect simple damage with limited accuracy, which may affect their subsequent repair performance.

In deep learning-based algorithms, although there are a few fully automatic repair methods [5,6] as mentioned previously, they are in the style of end-to-end repairing, which means that it is not easy to have control over the detection of damaged areas, potentially destroying or being unable to completely preserve valuable historical photos to the full degree. We note that although the image content is different between worn-out old photos and pavement crack images, the damage types are similar and both include mainly irregular cracks, so we review and discuss the related literature on pavement crack detection as well. König et al. [13] replaced standard convolutional blocks with residual blocks and added an attention gating mechanism to preserve spatial correlation in the feature map and suppress gradients in unrelated regions. Yang et al. [14] proposed a feature pyramid and hierarchical boosting network (FPHBN) to fuse features of different sizes. Lau et al. [15] used a pre-trained ResNet-34 to enhance the feature extraction capability of the network, while Liu et al. [16] used the dilated convolution approach to make the area of the receptive field wider.

It is mentioned in [17] that the ratio between cracked and non-cracked pavement is very imbalanced, often leading to poor network segmentation results and the failure of network training for crack detection, and a similar problem exists in our task. The solution to the imbalance between the cracked and non-cracked data can be adjusted by either the data set [15,17,18] or the loss function [15,16,19]. The dataset adjustment strategy breaks the picture into smaller blocks, such as 48 × 48, 64 × 64, or even multiple block sizes [15] for the training model, and then picks the proper ratio of cracked and non-cracked blocks for training to reduce the dataset imbalance problem. For example, Zhang et al. [17] used cracked blocks only as the training set for their crack-patch-only (CPO) supervised adversarial learning. Jenkins et al. [18] set the specific ratio between cracked and non-cracked blocks in the training set to place more weight on cracked blocks. As for the loss function, most works use binary cross-entropy (BCE) as a loss function for semantic segmentation-like applications, but this function is weak in handling the imbalanced dataset issue. As a consequence, Lau et al. [15] replaced BCE functions with dice coefficients to evaluate the correctness of the detected areas. Liu et al. [16] further combined the BCE functions and the dice coefficients. Cheng et al. [19] applied distance weight to improve the original binary cross entropy. Existing deep learning-based road crack detection algorithms can work on more complex and diverse damage than can traditional algorithms. However, since there are many differences between the content of road images and old photos, it is not possible to use the road crack detection method directly, so we need to develop a method suitable for detecting damage in old photos.

To summarize the main contributions of our work, unlike other literature approaches where the content of some intact areas is changed during repair, our way of recovering damaged old photos ensures no alteration of intact areas during repair to preserve photo integrity and fidelity. Since the existing methods for detecting image damage are not satisfactory, in this paper an automatic damage detection method is proposed for the recovery of old damaged photos to save time and effort. The advantage of our work is that our detection result enables the possibility of combining any subsequent inpainting methods to repair the photo, which is not possible using existing automatic end-to-end repairing methods.

## 2. Proposed Method

**Figure 1:**
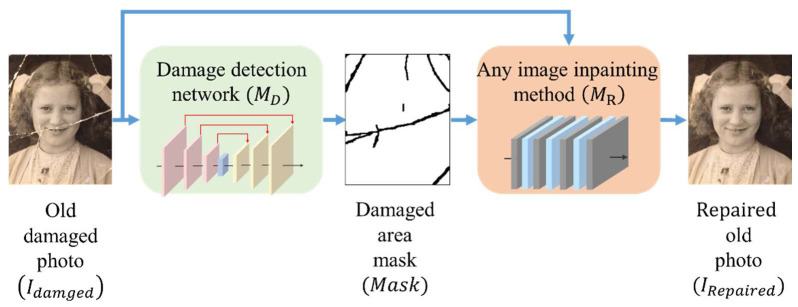
Flow chart of our architecture to automatically repair damaged old photos. By feeding an old damaged photo into our damage detection network, we can generate a damaged area mask. To restore the photo, the damaged photo and the mask are fed together into an arbitrary inpainting algorithm.

Our recovery processing of damaged old photos is divided into two parts, as shown in Figure 1. In the detection model ($M_{D}$), the model input is an old damaged photo ($I_{damaged}$) and the model output is a damaged area mask (Mask). The $I_{damaged}$ and the Mask are then exported to the inpainting method ($M_{R}$) to generate the repaired photo ($I_{Repaired}$), where the $M_{R}$ can be any existing method.

(1) $$Mask=M_{D}\left(I_{damaged}\right)$$

(2) $$I_{Repaired\;}=M_{R}\left(Mask,I_{damaged}\right)$$

**Figure 2:**
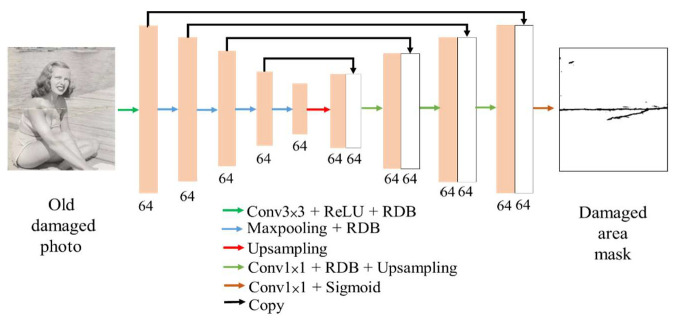
Architecture of the damage detection model.

Figure 2 shows the architecture of our damaged detection model is derived from U-Net [21]. The first half is an encoder that extracts the image features, while the second half restores the image to its original size by up-sampling and uses the sigmoid function to find out the map of pixel damage probabilities. The advantage of using U-Net is its ability to capture features at different scales, which are important for old photo damage detection and allow the model to more accurately identify damage in different shapes and sizes. Another merit of U-Net is the ability to concatenate features of the encoder into the decoder, allowing the model to train without losing the features obtained in the shallow network.

In order to improve the ability to extract features, we replaced the original convolutional layers of U-Net with residual dense blocks (RDBs) [22]. This block is a combination of a residual block [23] and a dense block [24]. The residual block uses a skip connection to combine the input of the block with the output of the block, thus increasing the stability of the model training and the speed of convergence. The dense block continuously passes all the shallow features of the block to the deeper layers, thus making full use of the information from the shallow features. The RDB retains these advantages to improve the performance of the whole model. The original convolution layers at each scale used in U-Net would gradually lose its shallow feature information, but this problem was solved when we adopted RDB. In this way, it is possible to use more information from the area surrounding the damage for damage detection.

**Figure 3:**
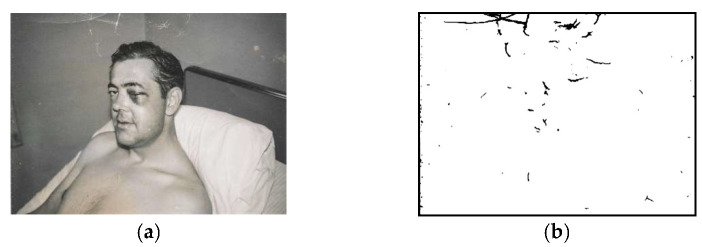
Dataset for damage detection: (**a**) old damaged photo; (**b**) corresponding marked ground truth.

Since there is no open dataset of damaged old photos available for use, we collected photos from the Internet and marked the damaged areas in the images by ourselves. These photos consisted mainly of portraits, buildings, and natural scenery, with their sizes ranging from 129 × 317 to 797 × 1131 pixels. To generate ground truths, we manually marked the damaged areas of the collected photos using the image editing tool GIMP [1]. The transparency function of the GIMP layer feature makes marking damaged areas in photos easier and more precise. Figure 3 shows examples of photos from our collected dataset as well as the corresponding marked ground truth. We collected a total of 170 old damaged photos and manually labeled them, 123 of which were for the training set, 18 for the test set, and the remaining 29 for the validation set. On account of the limited number of photos in the data collection, the data augmentation technique was used to increase the dataset size via horizontal flipping and the 90-, 180-, and 270-degree rotation of photos.

**Figure 4:**
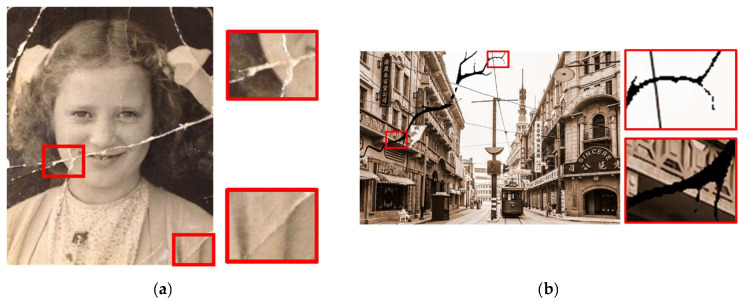
Real damaged photos and damaged photos synthesized by texture mask: (**a**) real damaged photo; (**b**) our synthesized damaged photo.

Because there are more undamaged old photos on the Internet, in order to further extend the training dataset we collected and used these undamaged photos, along with a collection of damage-like textures, to synthesize artificial damaged photos. Compared to Figure 4a we can see some differences between the artificially damaged photo and the old real damaged photo. The real damaged area of an old photo is composed of complex multitoned contents, not just simple good or bad, but our synthesized damaged photo only uses a single color to represent damage. We treated this difference as a type of damage to improve the generalizability of the model.

The model parameters are initialized using the MSRA initialization method [25] in the experiments, and the optimizer is the Adam optimizer with $\beta_{1}=$0.9, $\beta_{2}=$0.999. The initial learning rate of the model is set to 0.0001, and every 1000 epochs are multiplied by 0.1 to train a total of 2000 epochs. The training patch size 48 × 48, which is commonly used in pavement crack detection, is less appropriate for our task. The main reason for this is that most cracked pavement images only have a black background and a few white cracks, whereas old cracked photos have more complex content, such as portraits, objects, buildings, and so on. Therefore, we partitioned the photos of the training set into patches of 100 × 100 pixels in size to account for more context to improve the performance, and in our experiment, larger patch sizes than this did not result in any additional performance gain. We also controlled the ratio of patches with damaged areas to patches without damage at 8:2 in training.

The loss function was balanced cross entropy. The main reason for employing balanced cross entropy was to compensate for the imbalance between intact and damaged areas. It modified the original binary cross entropy with the ratio of the two categories, giving more weight to the fewer damaged areas and less weight to the more numerous intact areas as shown in (3) where N is the total number of pixels in training blocks, $\alpha_{i}$ is the weight of the intact areas, $y_{i}$ denotes whether the ith pixel belongs to the intact category in the ground truth, and $p\left(i\right)$ is the model’s prediction of the probability that the ith pixel belongs to the intact areas.

(3) $$L_{detection}=-\frac{1}{N}\sum_{i=1}^{N}\alpha_{i}\cdot y_{i}\cdot log\left(p\left(i\right)\right)+\left(1-\alpha_{i}\right)\cdot \left(1-y_{i}\right)\cdot log\left(1-p\left(i\right)\right),$$

## 3. Experiment Result

In our experiment, model training and testing were carried out on a computer equipped with an Intel i5-2400 CPU and an NVIDIA 2070 8GB GPU. To assess the model performance of damage detection, we adopted the evaluation methods commonly used in image segmentation and pavement crack segmentation, including precision, recall, F1-measure, and precision-recall curve (PR curve), as our evaluation metrics. Precision is the percentage of the results identified as damaged areas that are actually damaged. The percentage of true damaged areas detected is represented by recall. The F1 measure considers both precision and recall. Since the ground truth is created by manual marking and each person has different damage marking criteria, we adopted the regional precision and recall proposed in [26], which considers the detection result correct as long as it is within five pixels of the manual marking results, to compensate for the ground truth credibility problem caused by manual marking errors.

### 3.1. Comparison of Various Modules

**Figure 5:**
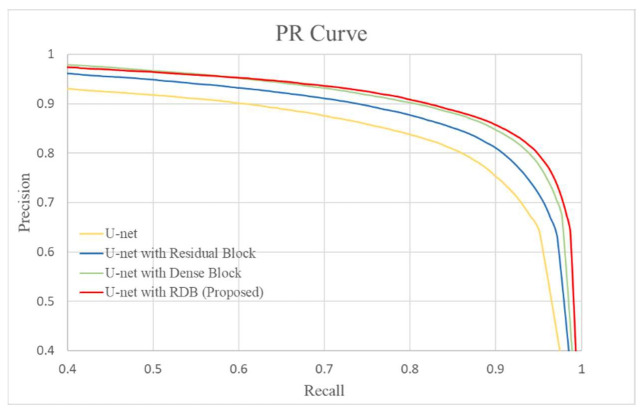
The PR curve of U-Net with various modules.

**Figure 6:**
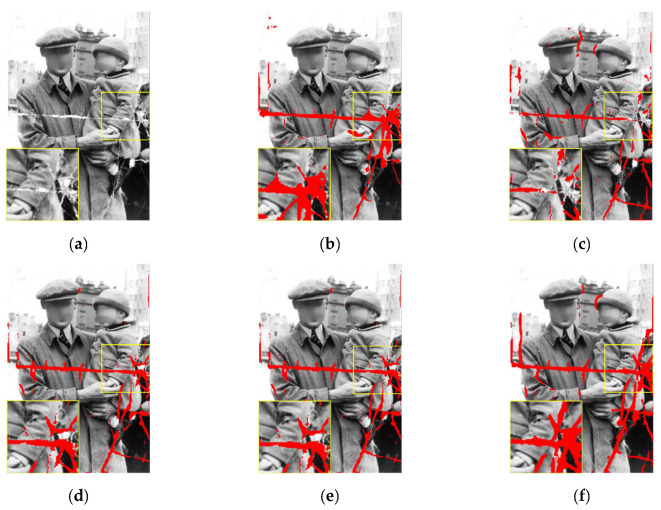
The detection results of different modules: (**a**) the old damaged photo; (**b**) labeled ground truth of damaged areas; (**c**) the detection result of U-Net; (**d**) the detection result of U-Net with residual block; (**e**) the detection result of U-Net with dense block; (**f**) our proposed detection result of U-Net with RDB.

In this section, we first evaluate the performance of our damage detection model on old photos by testing the performance of U-Net barebones combined with various modules. We compared the results of our proposed method with three methods, including the original U-Net architecture, the U-Net architecture with a residual block module, and the U-Net architecture with a dense block module. Figure 5 and Table 1 show the results in terms of the PR curve, precision, recall, and F1-measure, which show that our proposed approach outperformed all other module combinations. Figure 6 depicts the visual outcome of using various modules to detect damage. It can be seen that our proposed method is capable of detecting more subtle damage as well as the damage border. The more complete the detection, particularly along the damage border, the more it can assist us in repairing damage without affecting the repair result.

### 3.2. Comparison of Different Detection Methods

**Figure 7:**
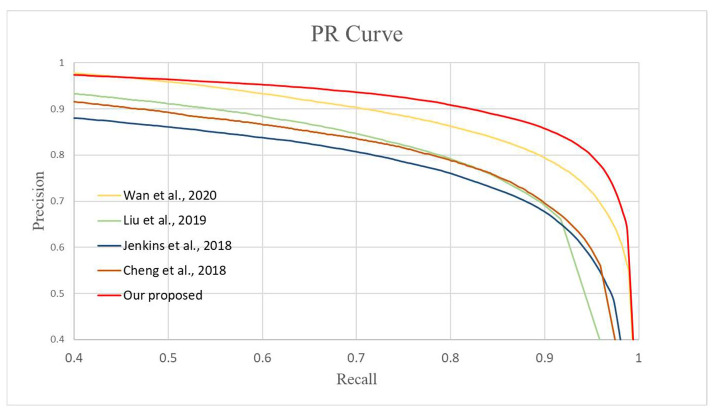
The PR curve of different methods, including [5,16,18,19], and our proposed method.

Next, we compare our method with other methods in the literature. We disassembled the damage detection part from the whole end-to-end work [5] and compared it to our method. Since there are so few existing deep learning-based damage detection methods for old photos, we also compared the results of pavement crack detection models [16,18,19] that have been retrained using our dataset to work on old photo damage detection. The results of the PR curve are shown in Figure 7. The best recall, precision, and F1 measure values for each method are shown in Table 2. The comparison results show that our detection effect is the best.

**Figure 8:**
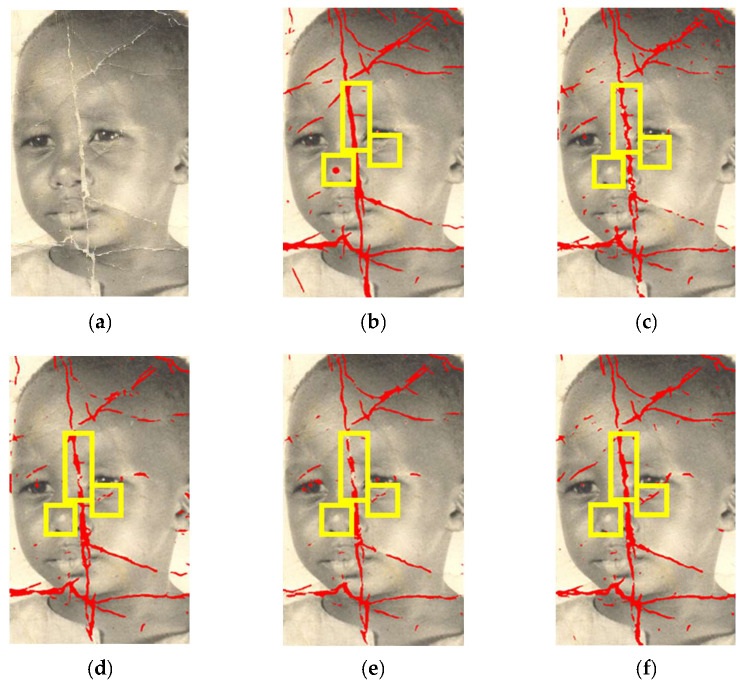
The results of different detection methods, with yellow boxes indicating areas of performance difference: (**a**) the old damaged photo; (**b**) the result of Wan et al. [5]; (**c**) the result of Liu et al. [16]; (**d**) the result of Jenkins et al. [18]; (**e**) the result of Cheng et al. [19]; (**f**) the result of our proposed method.

Figure 8 compares the visual results of the proposed method with those detected by other methods. It can be seen that our proposed method of detecting damage in the photo was more accurate, especially in the detection border denoted inside the yellow boxes. By contrast, the methods proposed by [16,18,19] failed to completely detect the damage in the image, and [5] often labeled undamaged areas as damage, such as around the tip of the nose in Figure 8.

As shown the Table 3, we also compared the number of parameters and computation speed with these methods [5,16,18,19] where the size of the test photos was 512 × 512. Jenkins et al. [18] and Cheng et al. [19] used the same model framework, but the model was trained using different strategies. Therefore, they have the same number of parameters and running time. Table 3 shows that both our detection models and those of [5] are fast as both lower to the scale of $10^{-3}$ s, but our model is much lighter as our number of parameters is only about one-sixteenth of all the other methods.

### 3.3. Combination with Inpainting Methods

**Figure 9:**
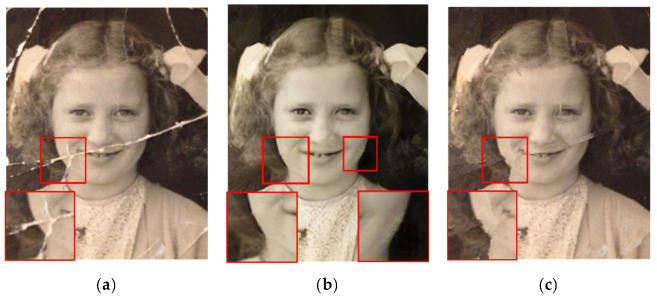

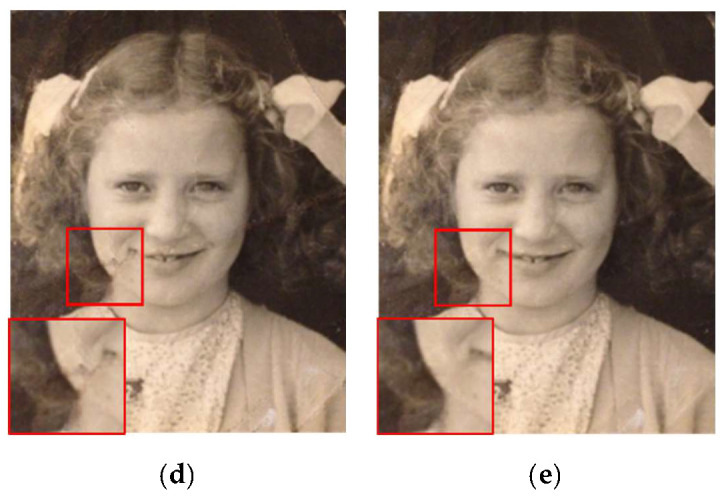
Results of different restoration methods on the damaged photo: (**a**) the old damaged photo; (**b**) the result of Wan et al. [5]; (**c**) the result of ours + Yu et al. [27]; (**d**) the result of ours + gated convolution [4]; (**e**) the result of ours + partial convolution [28].

**Figure 10:**
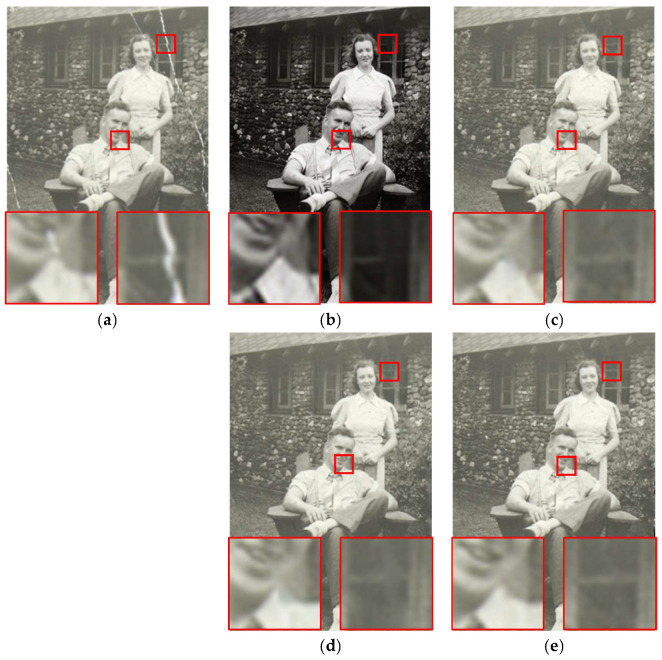
Results for different restoration methods on the damaged photo: (**a**) the old damaged photo; (**b**) the result of Wan et al. [5]; (**c**) the result of ours + Yu et al. [27]; (**d**) the result of ours + gated convolution [4]; (**e**) the result of ours + partial convolution [28].

Next, we present our results regarding practical application. We used [4,27,28] as the inpainting method in the subsequent process to repair actually damaged photos. The repair results using actually damaged photos are shown in Figure 9c–e and Figure 10c–e, which demonstrate the results of our damage detection followed by different inpainting methods [4,27,28]. We can see in Figure 9c that Yu [27] failed to repair the cheeks and mouth in our detected area. Repair to damaged areas by gated convolution [4] is generally blurred as shown in Figure 9d. Figure 10c,d shows that deformation of the collar edge occurred after restoration. In general, the results of partial convolution [28] as shown in Figure 9e and Figure 10e are more satisfactory compared to other inpainting methods [4,27]. This demonstrates that our architecture can be combined with any inpainting method, but we suggest that partial convolution [28] will achieve better results. In Figure 9b and Figure 10b, we also compare our method with the end-to-end method [5], which integrates damage detection and repairs in one stage. Although [5] looks to have been effective in repairing the damaged areas, there are some color distortion problems with unfaithful tonal changes and a loss of texture in the image, such as in the cheeks as shown in Figure 9b. We can see that there are unrestored damaged areas and missing window frame details marked in the red box in Figure 10b. Thus, combining our architecture with the inpainting method [4,27,28] in contrast to [5] provides better results without affecting content in the undamaged regions in the recovery results.

**Figure 11:**
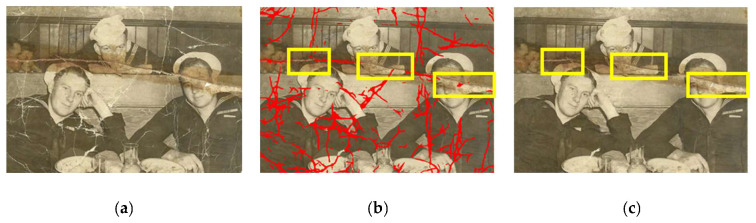
The case of failure detection: (**a**) damaged photo; (**b**) result of damage detection; (**c**) result of damage restoration.

There will still be cases where our approach may fail. For example, if the model encounters a mixture of various complex damage, as shown in Figure 11, it becomes difficult to distinguish the damaged areas, resulting in partial detection and incomplete repair results. To deal with such a complex pattern of damage, future studies could investigate and apply the concept of directional clues in damage patterns [29,30,31] to aid in crack damage detection.

## 4. Conclusions

Most restoration methods for damaged old photos require the manual marking of damaged areas for restoration, which is quite inefficient. Therefore, we proposed a damage detection model for old photos. Our method can detect damaged areas automatically without manual marking, which significantly reduces repair time. The detection results can be optionally screened and flexibly combined with any powerful inpainting method to fully automatically recover the content of the photos. We analyzed various block modules to design the detection model and found that the residual dense block (RDB), which combines the advantages of residual block and dense block, can effectively improve model detection capability. When compared to other detection algorithms, our method can detect damaged areas more accurately. We demonstrated the restoration of damaged old photos by combining our detection results with three different inpainting methods. In our restoration results, both the damaged and undamaged areas of the photos did not suffer from color tone changes, color distortion, or texture loss. Our method can better preserve the integrity of photos than can the existing end-to-end method, which alters the undamaged areas of photos.

## Figures and Tables

**Table 1:** The recall, precision, and F1 measure of different modules.

Structure	Recall	Precision	F1 Measure
U-Net	0.857	0.802	0.817
U-Net with residual block	0.876	0.833	0.846
U-Net with dense block	0.903	0.843	0.866
U-Net with RDB (proposed)	0.911	0.847	0.873

**Table 2:** The recall, precision, and F1 measure of different methods.

Method	Recall	Precision	F1 Measure
Wan et al. [5]	0.845	0.837	0.831
Liu et al. [16]	0.832	0.767	0.785
Jenkins et al. [18]	0.838	0.734	0.763
Cheng et al. [19]	0.839	0.763	0.784
Our proposed method	0.911	0.847	0.873

**Table 3:** Parameter and run time.

Method	Parameter	Computation Time (s)
Our proposed method	2.3 M	0.0084
Wan et al. [5]	37 M	0.0042
Liu et al. [16]	31.38 M	0.0122
Jenkins et al. [18]	33.24 M	0.0162
Cheng et al. [19]	33.24 M	0.0162

## Data Availability

Some damaged source images were obtained from https://commons.wikimedia.org/wiki/Category:Damaged_photographs#/media/File:1945BunnyLakeTeeth.jpg under CC BY-SA 2.0 license, as well as from https://www.flickr.com/photos/simpleinsomnia/25293432854/in/photostream/ under CC BY 2.0 license.
